# Novel *Chlamydia trachomatis* Strains in Heterosexual Sex Partners, Indianapolis, Indiana, USA

**DOI:** 10.3201/2011.140604

**Published:** 2014-11

**Authors:** Byron E. Batteiger, Raymond Wan, James A. Williams, Linda He, Arissa Ma, J. Dennis Fortenberry, Deborah Dean

**Affiliations:** Indiana University School of Medicine, Indianapolis, Indiana, USA (B.E. Batteiger, J.A. Williams, J.D. Fortenberry);; Children’s Hospital Oakland Research Institute, Oakland, California, USA (R. Wan, L. He, A. Ma, D. Dean);; University of California, Berkeley, California, USA (D. Dean);; University of California, San Francisco, California, USA (D. Dean)

**Keywords:** Chlamydia trachomatis, MLST, partner tracing, recombinant strains, bacteria, STIs, sexually transmitted infections, heterosexual partners, Indiana, USA

## Abstract

*Chlamydia trachomatis* causes a high number of sexually transmitted infections worldwide, but reproducible and precise strain typing to link partners is lacking. We evaluated multilocus sequence typing (MLST) for this purpose by detecting sequence types (STs) concordant for the *ompA* genotype, a single-locus typing standard. We tested samples collected during April 2000–October 2003 from members of established heterosexual partnerships (dyads) in the Indianapolis, Indiana, USA, area who self-reported being coital partners within the previous 30 days. *C. trachomatis* DNA from 28 dyads was tested by MLST; sequences were aligned and analyzed for ST and phylogenetic relationships. MLST detected 9 *C. trachomatis* STs, 4 unique to Indianapolis; STs were identical within each dyad. Thirteen unique strains were identified; 9 (32%) dyads harbored novel recombinant strains that phylogenetically clustered with strains comprising the recombinants. The high rate of novel *C. trachomatis* recombinants identified supports the use of MLST for transmission and strain diversity studies among at-risk populations.

## Abstract 2

Use of multilocus sequence typing may help identify new strains in at-risk populations.

*Chlamydia trachomatis*, a bacterium that can infect both men and women, is most commonly sexually transmitted. In 2008, approximately 105.7 million new *C. trachomatis* sexually transmitted infections (STIs) occurred worldwide (*1*); an estimated 2.86 million incident cases occurred in the United States (*2*). The last surveillance study of STIs in the United States, in 2011, reported 1,412,791 chlamydial infections, the largest case number for any disease ever reported to the Centers for Disease Control and Prevention (*3*). 

*C. trachomatis* infections in men and women are mostly asymptomatic; thus, continued sexual activity among persons unaware of their infection status facilitates further transmission. Gaps in knowledge of chlamydial STIs include how measures of immunity, bacterial load, condom use, and other factors relate to transmission risk. Longitudinal studies of these factors are needed to inform treatment and prevention strategies (*4*). The tools required include careful ascertainment of sexual history and behavioral determinants and reproducible and discriminating biomarkers to strengthen the case for transmission between sex partners linked by partner tracing.

The standard biomarker for these studies is *ompA* genotyping, but this method lacks precision because the gene is under immune selection and represents only 0.1% of the genome. Because large-scale whole-genome sequencing of clinical samples is not yet feasible, multilocus sequence typing (MLST) for *C. trachomatis* has been developed to provide greater insight into strain types; 3 such MLST methods have been reported in the literature (*5*–*10*). The scheme we developed, on the basis of analysis of 19 reference strains and 68 geographically diverse clinical isolates, identified 44 MLST sequence types (STs), compared with only 20 *ompA* genotypes (*11*). In our scheme, we were also able to discriminate single-nucleotide polymorphisms (SNPs) that correlate with disease phenotypes attributable to *C. trachomatis*: lymphogranuloma venereum (LGV), trachoma, and non-LGV urogenital diseases (*11*). Our scheme has since been expanded to encompass 192 geographically and clinically diverse samples.

For this study, we applied our MLST scheme to a subset of a well-defined heterosexual partnership (dyad) cohort in Indianapolis, Indiana, USA, comprising 28 dyads for which concordance of the *ompA* genotype existed between partners. The purpose of the study was to determine whether MLST, which provides a more detailed level of strain typing than *ompA* genotyping, would also show strain concordance between partners, as would be expected if transmission had occurred within the dyads. In addition, we sought to identify additional *C. trachomatis* strain types, beyond those identified by *ompA* genotyping, that might be unique to Indianapolis, because this geographic region has not previously been included in any MLST database.

## Materials and Methods

### Study Population

A study of *C. trachomatis* concordance in heterosexual partnerships (dyads) was conducted in Indianapolis during April 2000–October 2003; participants were sexually active heterosexual men and women 15–25 years of age who visited an urban STI clinic (*12*). Written informed consent was obtained, and the study was approved by the Indiana University–Purdue University Institutional Review Board. Eligibility was defined as self-reported sexual activity between the partners during the previous 30 days. A total of 210 heterosexual dyads were established by research disease intervention specialists and enrolled. *C. trachomatis* infection was identified by Amplicor CT/NG (Roche Diagnostics, Indianapolis, IN, USA) nucleic acid amplification test and cell culture, as previously described (*12*). Of the 210 dyads, 130 contained >1 *C. trachomatis*–infected partner; for 45 dyads, both partners were infected and had identical *ompA* genotypes. 

For the MLST study, we used remainder samples from 56 members of 28 dyads who were concordant for *C. trachomatis* infection and *ompA* genotype. These samples were provided to investigators at Children’s Hospital Oakland Research Institute (CHORI) in a de-identified and blinded fashion. Thus, CHORI research was considered not to involve human subjects, and informed consent was not required.

### Reference and Clinical Samples

We used 56 samples (from cervix in women and urethra in men) from 28 dyads in which persons within each dyad were concordant for *C. trachomatis* infection and *ompA* genotype. Additionally, we used for analysis MLST data for 20 *C. trachomatis* reference strains (A/Sa1, A/HAR13, B/TW5/OT, Ba/Apache2, C/TW3/OT, D/UW3/Cx, Da/TW448, E/Bour, F/ICCal3, G/UW57/Cx, H/UW4/Cx, I/UW12/Ur, Ia/UW202, J/UW36/Cx, Ja/UW92, K/UW36/Cx, L_1_/440, L_2_/434, L_2_a/UW396, L_3_/404) and 172 clinical samples in the MLST database (http://www.mlst.net).

### *ompA* Genotyping and MLST Analyses

*ompA* genotyping of the samples had been previously performed at Indiana University (*13*,*14*) as part of the earlier *C. trachomatis* concordance study. Cultured and noncultured clinical samples were sent to CHORI for analysis (*11*). DNA was extracted, and MLST for 7 housekeeping genes was performed by using primers as described (*11*; http://www.mlst.net; Technical Appendix Table 1). A consensus sequence was created from forward and reverse sequences, and the genes were concatenated and queried against all 202 MLST sequences in the database (*15*). Sequence output was used to identify each unique allelic profile to assign an ST, and all STs were deposited in the *C. trachomatis* database (http://chlamydia.mlst.net). The concatenated sequences of the 7 MLST loci and the allelic profiles for each sample were used to identify sample relatedness.

*ompA* genotypes were defined on the basis of homology with reference strains of *C. trachomatis*. If >1 SNP was identified when sequences were compared to those of the closest hit reference strain, a number was used to denote the presence of the SNP(s) (e.g., Ia4) (*15*).

### Strain Clustering and SNP Analyses

Strain clustering and SNP analyses were performed as described (*11*). Briefly, clusters of related and singleton STs as well as evolutionary patterns among the isolates and for the entire dataset were determined by using eBURST (http://eburst.mlst.net). Neighbor-joining and minimum evolution methods in MEGA4 (http://www.megasoftware.net) were used to construct the trees along with multiple substitution models, including p-distance and Jukes-Cantor; all methods gave similar results. To test support for each node in the tree, we performed 1,000 bootstrap replicates.

All SNPs were identified for each ST by using the PROC FREQ tool in SAS software (SAS Institute, Cary, NC, USA). The probability of association of a SNP with an ST was determined by using a classification index (*16*). Variance across the dataset was determined using the Levene test (*17*). A p value <0.05 was considered significant.

## Results

### MLST Discrimination of *C. trachomatis*

The Table shows the distribution of MLST STs and *ompA* genotypes for each dyad with SNP location(s), if present, for each of the 7 MLST housekeeping genes. We noted that in some cases, the DNA extracted directly from the patient sample was sufficient for MLST, whereas in other cases, the cultured sample was required because the patient sample did not yield sufficient DNA for MLST. We found no differences in MLST results when we compared DNA directly extracted from the patient sample with DNA extracted from the culture of the same sample.

**Table:** *Chlamydia trachomatis* *ompA* genotypes, MLSTs, and SNPs for samples from heterosexual patient pairs (dyads) in Indianapolis, Indiana, USA, April 2000–October 2003*

Dyad no.	Sample nos.	*ompA* genotype	ST	*ompA* genotype(s) associated with ST–closest hit genotype homology (SNPs)†
1	**J/112i**	**J/UW36/Cx**	**15**	**J/UW36/Cx & K/UW36/Cx–K/42nl & K/49nl **
	**J/113i**			
2	K/186i	K/UW36/Cx	15	J/UW36/Cx & K/UW36/Cx–K/42nl & K/49nl
	K/187i			
3	**H/114i**	**H/UW4/Cx**	**19**	**D/UW3/Cx, G/UW57/Cx, H/UW4/Cx, I/UW12/Ur, J/UW36/Cx–G/SotonG1 **
	**H/115i**			
4	Ia/94i	Ia/UW202	23	D/UW3/Cx, Ia/UW202–Ia/UW202
	Ia/95i			
5	Ia/118i	Ia/UW202	23	D/UW3/Cx, Ia/UW202–Ia/UW202
	Ia/119i			
6	Ia4/177i	Ia4	23	D/UW3/Cx, Ia/UW202, Ia4–Ia/UW202
	Ia4/180i			
7	Ia/178i	Ia/UW202	23	D/UW3/Cx, Ia/UW202–Ia/UW202
	Ia/179i			
8	Ia/183i	Ia/UW202	23	D/UW3/Cx, Ia/UW202–Ia/UW202
	Ia/184i			
9	**D2/96i**	**D2**	**34**	**D2, D/UW3/Cx, E/Bour, F/ICCal3, Ja/UW92–F/ICCal3 **
	**D2/97i**			
10	F/98i	F/ICCal3	34	D2, D/UW3/Cx, E/Bour, F/ICCal3, Ja/UW92–F/ICCal3
	F/99i			
11	F/181i	F/ICCal3	34	D2, D/UW3/Cx, E/Bour, F/ICCal3, Ja/UW92–F/ICCal3
	F/182i			
12	**D2/189i**	**D2**	**34**	**D2, D/UW3/Cx, E/Bour, F/ICCal3, Ja/UW92–F/ICCal3 **
	**D2/190i**			
13	F/191i	F/ICCal3	34	D2, D/UW3/Cx, E/Bour, F/ICCal3, Ja/UW92–F/ICCal3
	F/192i			
14	E/88i	E/Bour	39	E/Bour–E/Bour
	F/89i			
15	E/102i	E/Bour	39	E/Bour–E/Bour
	E/103i			
16	E/106i	E/Bour	39	E/Bour–E/Bour
	E/107i			
17	E/116i	E/Bour	39	E/Bour–E/Bour
	E/117i			
18	E6/120i	E/Bour	39	E/Bour–E/Bour
	E6/121i			
19	E/108i	E/Bour	39	E/Bour–E/Bour
	E/109i			
20	E/110i	E/Bour	39	E/Bour–E/Bour
	E/111i			
21	E/171i	E/Bour	39	E/Bour–E/Bour
	E/172i			
22	**D1/90i**	**D1**	**45**	**D1–F/ICCal3 (*glyA:* 176, 264)**
	**D1/91i**			
23	**E/92i**	**E/Bour**	**46**	**E/Bour–Da/TW448 (*leuS:* 58, 96)**
	**E/93i**			
24	**E/104i**	**E/Bour**	**46**	**E/Bour–Da/TW448 (*leuS:* 58, 96)**
	**E/105i**			
25	**E/173i**	**E/Bour**	**46**	**E/Bour–Da/TW448 (*leuS:* 58, 96)**
	**E/174i**			
26	**E/185i**	**E/Bour**	**46**	**E/Bour–Da/TW448 (*leuS:* 58, 96)**
	**E/188i**			
27	E/100i	E/Bour	47	E/Bour–E/Bour (*hybG:* 257, 289, 451; *pykF:* 317, 384)
	E/101i			
28	F4/175i	F4	55	F4–F/ICCal3 (*leuS*: 44)
	F4/176i			

*Boldface indicates putative recombinant strains. MLST, multilocus sequence typing; SNP, single-nucleotide polymorphism; ST, sequence type. †*ompA* genotypes that are associated with each ST are listed; after the dash, the genotype that is the closest hit for the 7 MLST housekeeping genes is listed. In some cases, the closest hit is a reference strain with SNPs in a gene; the location of the SNP is listed based on the start position of the gene as designated by the genome sequence of D/UW3/Cx (GenBank accession no. AE001273.1).

For each of the 28 dyads, both partners had the same MLST ST. A total of 9 STs were found in the 56 samples from the 28 dyads: ST15, ST19, ST23, ST34, ST39, ST45, ST46, ST47, and ST55. Twenty-one dyads harbored MLST STs that matched STs of samples obtained from geographically diverse areas currently included in the MLST database, whereas 7 dyads harbored newly identified STs not present in the database (Technical Appendix Table 2). Among these 7 dyads, 4 MLST STs were unique to Indianapolis: ST45 (dyad 22), ST46 (dyads 23–26), ST47 (dyad 27), and ST55 (dyad 28); these results reflect the SNPs in various alleles (Table).

Eleven *ompA* genotypes were represented in the study and, by study design, were identical within dyads. The *ompA* genotypes included E, F, H, Ia, J, and K sequences that were identical to those of reference strains and 5 variant *ompA* genotypes of D1, D2, E6, F4, and Ia4 that had SNPs compared with the reference strains.

By combining MLST ST and *ompA* genotype data, we identified 13 unique strains (9 by MLST and 4 by *ompA* genotype) among the samples from our study group. Among these 13 strains, 8 were unique to Indianapolis and were found in 12 dyads (dyads 1, 6, 9, 12, 18, 22, 23, 24, 25, 26, 27, and 28) (Table; Technical Appendix Table 2). Moreover, of the 13 strains identified among the dyad samples, 9 (69%) contained gene sequences that suggested recombination within the genome, meaning that the *ompA* genotype was different from the *ompA* genotype that should be associated with the MLST ST if the genome were just 1 strain. For example, for strains from dyads 10 and 12, the *ompA* was D2, but the sequences of the 7 housekeeping genes (MLST ST34) matched the 7 housekeeping genes of strain F/ICCal3 from the MLST database. Putative recombinants (boldface in Table) represented a rate of 32% (9 of 28 samples).

MLST STs and *ompA* genotypes for each sample in the MLST dataset are shown in Technical Appendix Table 2. We found substantial variability of *ompA* genotypes associated with MLST STs, as shown previously (*11*). For the STs for the 56 Indianapolis samples, ST15 was associated with *ompA* genotypes J and K; ST34 was associated with *ompA* genotypes D2, E, and F; and ST19 was associated with *ompA* genotypes D, G, H, I, and J. online Technical Appendix Table 3 shows the characteristics of the alleles for each MLST locus based on the inclusion of the Indianapolis dataset in the MLST database.

### Phylogeny of STs by Disease Phenotype and Evidence for Recombination

**Figure 1:**
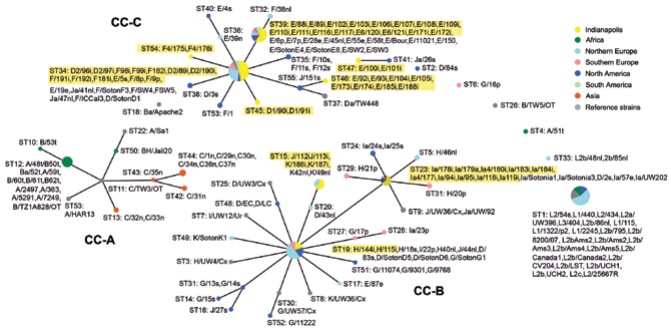
Population snapshot for *Chlamydia trachomatis* samples collected during April 2000–October 2003 from members of heterosexual partnerships (dyads) in Indianapolis, Indiana, USA, compared with reference strains. Data were compiled in eBURST (http://www.mlst.net). Three distinct clonal complexes (CCs) are shown, along with numerous singletons of various sizes and 1 doublet. CC-A, strains causing trachoma; CC-B, noninvasive, nonprevalent urogenital strains; CC-C, noninvasive, globally prevalent urogenital strains. Samples from Indianapolis are highlighted in yellow (shown with sample identification number) and are restricted to clusters I and III. Each circle represents a sequence type (ST) at the point where linked STs within each CC are likely to have descended from the same recent ancestor. The area of the circle denotes the number of samples for that ST. The primary founder of the CC is at the hub; subgroup founders are represented as secondary hubs (e.g., C/35n).

The association of disease phenotype with 3 clonal complexes (CCs) was identified by eBURST (Figure 1), similar to those we reported previously: *C. trachomatis* strains that cause trachoma A, B, Ba, and C (CC-A); noninvasive STIs with low population prevalence (CC-B); and noninvasive, globally prevalent D/Da, E, and F STIs (CC-C). The Indianapolis strains were confined to noninvasive STI CCs (B and C), as expected. The strains associated with 3 of the 4 unique STs in the Indianapolis samples are seen in CC-C (Figure 1).

**Figure 2:**
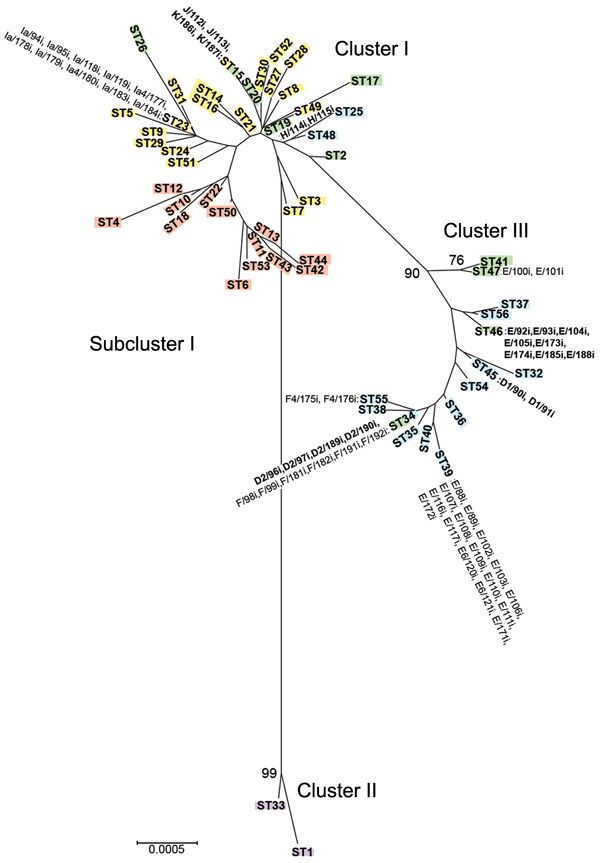
Minimum evolution tree of *Chlamydia trachomatis* samples collected during April 2000–October 2003 from members of heterosexual partnerships (dyads) in Indianapolis, Indiana, USA, compared with reference strains. The tree was constructed by using the 192 concatenated sequences in the MLST database (http://www.mlst.net) for the 7 loci. Bootstrap values (1,000 replicates) >70% are shown. Three clusters and 1 subcluster are shown: cluster I, yellow, noninvasive, nonprevalent sexually transmitted infection (STI) strains; subcluster I, red, trachoma strains; cluster II, purple, invasive lymphogranuloma venereum strains; and cluster III, blue, noninvasive, highly prevalent STI strains. Green denotes putative recombinant stains. Samples from Indianapolis are indicated next to sequence types; those in boldface are putative recombinants. Scale bar indicates number of substitutions per site.

The minimum-evolution tree also displayed 3 disease clusters (Figure 2); each of the Indianapolis strains is denoted next to the corresponding ST. Cluster I grouped noninvasive, low-prevalence STIs (eBURST CC-B), including a subcluster of strains that cause trachoma (eBURST CC-A). Cluster II grouped only invasive LGV strains. Cluster III grouped noninvasive, prevalent D/Da, E, and F STIs (eBURST CC-C). The tree constructed based on amino acid analysis showed similar clustering (data not shown).

The 9 putative Indianapolis recombinants were localized on the MLST tree with strains of the same *ompA* genotype and were recombinants of strains within the same cluster. Most recombinants were in cluster III. Four dyads with ST46 (unique to Indianapolis) had *ompA* genotype E and homology of the 7 housekeeping genes to reference strain Da/TW448 but with SNPs in *leuS*. D1/90i and D1/91i (ST45) and D2/96i, D2/97i, D2/189i, and D2/190i (ST34) were recombinants with homology to F/ICCal3 with SNPs in *glyA* and F/ICCal3, respectively. In cluster I, J/112i and J/113i shared the same ST as K/186i and K/187i and were recombinants with K/42nl and K/49nl. H/114i and H/115i were recombinants with G/SotonG1.

## Discussion

We investigated a well-defined, epidemiologically linked partner cohort of persons with *C. trachomatis* infection in which members of each dyad shared strains with identical *ompA* genotype and found that MLST ST was identical as well. This study confirms the reproducibility of MLST and short-term (≈30 days) stability of MLST in the context of a sexual partnership in which transmission has likely occurred. Whereas the identification of 8 unique *C. trachomatis* strains in Indianapolis was not surprising, given that samples from this city had not previously been subjected to MLST, the rate of 32% (9/28 samples) for recombinants was striking. We only considered 28 dyads in our analyses of strain diversity because of the closely defined epidemiologic link within the partnerships and the fact that strains were identical within dyads.

Becuase MLST provides >3 times the genetic data of *ompA*, the additional discriminatory power of this typing method is not surprising. We found that 8 of the 28 Indianapolis dyads (dyads 1 and 22–28) contained *ompA* genotypes that did not match our previous associations of *ompA* genotype with MLST STs in the MLST database (Table). For example, a J strain by *ompA* genotyping was associated with the MLST ST of a K strain (dyad 1); an *ompA* E strain was associated with the MLST ST of a Da strain (dyads 23–26); an *ompA* D1 strain was associated with the MLST ST of an F strain with SNPs in *glyA* (dyad 22); an *ompA* E strain was associated with the MLST ST of an E strain with 3 SNPs in *hybG* and 2 in *pykF* (dyad 27); and an *ompA* strain F4 matched the MLST of an F strain with an SNP in *leuS* (dyad 28). 

*ompA* genotyping should not be considered a formal part of an MLST scheme because it is under immune selection (*18*), and housekeeping genes provide a stable evolutionary marker for STs. However, *ompA* genotyping remains a useful tool because it has been the mainstay of typing *C. trachomatis* for >20 years and is valuable for comparison with strains typed only by this method. Furthermore, *ompA* genotyping, but not MLST, was able to identify 2 dyads in which partners were infected with strains exhibiting mutations in *ompA* that had not previously been detected: E6 (dyad 18) and Ia4 (dyad 6) (Table). This result indicates utility in continuing *ompA* genotyping as a separate but adjunctive method with MLST for epidemiologic and transmission studies and for establishing strain concordance among members of less well-defined partnerships.

We further identified 3 clonal complexes that correlated with phenotypic disease, similar to previous findings (*11*). Most Indianapolis samples clustered with noninvasive D/Da, E, and F strains in CC-C (Figure 1), a result that is expected, given that these strains are the most prevalent worldwide (*19*–*22*). Whereas the Indianapolis samples were represented in 9 STs, 4 of these STs were distinct for this city, which suggests some clonal expansion of those unique strains in this area.

Genomic characteristics may also drive specific events, such as recombination, that may result in clonal expansion within a relatively small sexual network. Recombinants of the most prevalent urogenital *ompA* genotypes E, F, and D have previously been reported (*23*,*24*). Several reports have also been published regarding recombinants between genotypes D, E, and F and *ompA* genotype J (*11*,*24*,*25*); recombinants of LGV and D strains have also been documented (*6*,*10*,*23*), and previous MLST studies have shown evidence for recombination (*7*,*10*,*11*). In a previous study, we found 9 (17%) of 53 urogenital samples, excluding all ocular samples from patients with trachoma, were recombinant among a geographic distribution that included the western United States, Portugal, the Netherlands, and Ecuador (*11*)*.*

In this study, members of 9 (32%) of the 28 dyads were infected with strains that contained gene sequences suggesting recombination within the genome (Table); this was the case for 2 of 4 STs that were unique to Indianapolis (STs 45 and 46). The 9 putative recombinants were localized on the MLST tree with strains of the same *ompA* genotype and, not surprisingly, were recombinants of strains within the same cluster (Figure 1). Our sample size was small, but the high rate of recombination suggests emerging diversity within a tight sexual network. This hypothesis is supported by historic studies of *ompA* genotypes among patients attending inner city STD clinics; in one such example, Ia genotypes that have much lower prevalence in other parts of the United States predominated among patients in Birmingham, Alabama, and were more prevalent than genotype D, which was the third most prevalent genotype for all other cities studied (*26*).

Our study has several strengths. Availability of the concordance study with carefully defined sexual partnerships, application of MLST to confirm the concordance of samples between dyads and to identify unique strains, and use of full-length *ompA* sequences enabled us to identify *ompA* variants and compare and combine the results of the 2 strain typing methods. The weaknesses of our study include the relatively small numbers of sexual partners and that only concordant dyads with epidemiologically linked strains were studied, limiting our conclusions about the overall diversity of strains in Indianapolis, which are likely much larger than what we discovered here.

In summary, our findings validate the discriminatory power of MLST for partnership and transmission studies of *C. trachomatis* infections among at-risk populations locally and globally. Larger partner and population studies that use MLST and *ompA* genotyping will provide valuable data on transmission concordance or discordance that will inform interventions and public health policy to better control *C. trachomatis* transmission. Furthermore, applying these tools globally will expand our knowledge of *C. trachomatis* strain diversity and their emergence among populations at risk for chlamydial STIs.

Technical Appendix Primer pairs used for PCR of the 7 multilocus sequence typing housekeeping genes for *Chlamydia trachomatis,* sequence types and characteristics of reference and clinical strains, and characteristics of alleles for each locus.
