# Supplementary material for: Novel Chlamydia trachomatis Strains in Heterosexual Sex Partners, Indianapolis, Indiana, USA
Source: Emerg Infect Dis. 2014 Nov;20(11):1841–7. doi: 10.3201/2011.140604 (PMC4214310; doi:10.3201/2011.140604)
Supplement: Technical Appendix — Primer pairs used for PCR of the 7 multilocus sequence typing housekeeping genes for Chlamydia trachomatis, sequence types and characteristics of reference and clinical strains, and characteristics of alleles for each locus. [file 14-0604-Techapp-s1.pdf]

Article DOI: http://dx.doi.org/10.3201/eid2011.140604

Novel *Chlamydia trachomatis* Strains in Heterosexual Sex Partners, Indianapolis, Indiana, USA

Technical Appendix

Technical Appendix Table 1. Primer pairs used for PCR of the 7 multilocus sequence typing housekeeping genes for *C. trachomatis*

Locus	Region	Primer name	Sequence, 5′ → 3′	Sequence length, bp
glyA	CT432	FglyA	GAAGACTGTGGCGCTGTTTTATGG	522
		RglyA	CTTCCTGAGCGATCCCTTCTGAC	
		Alternate:		
		PCRFa	GAACATAAGCCCACCGTTCT	
		PCRRa	TTCCAGATCGATTTCAGGAT	
mdhC	CT376	FmdhC	GGAGATGTTTTTGGCCTTGATTGT	519
		RmdhC	CGATTACTGCACTACCACGACTCT	
		Alternate:		
		PCRFa	AGGGCAAATAGCCTATAGCT	
		PCRRa	AAGCTCGTGCTGCAGAAGCT	
pdhA	CT245	FpdhA	CTACAGAAGCCCGAGTTTTT	549
		RpdhA	CTGTTTGTTGCATGTGGTGATAA	
		Alternate:		
		PCRFa	CATCCTCTGACTCTCAACAT	
		PCRRa	TAGGATCGGAAATAGAGTGT	
yhbG	CT653	FyhbG	TCAAGTCAATGCAGGAGAAAT	504
		RyhbG	GATAGTGTTGACGTACCATAGGAT	
		Alternate:		
		FPCR-LH	AATGATGTGTCCTTTCAAGT	
		RPCR-LH	AGAGTCTCCTAGATAGTGTT	
pykF	CT332	FpykF	ATCTTATCGCTGCTTCGTT	525
		RpykF	CAGCAATAATAGGGAGATA	
		Alternate		
		FpykF2	ACTTAAAATTTGGGGTAGAAC	
		RpykF2	ACAGCTAAACGATAGTACACAT	
lysS	CT781	FlysS	GAAGGAATCGATAGAACGCATAAT	576
		RlysS	ATACGCCGCATAACAGGGAAAAAC	
		Alternate:		
		FPCR2	GAATGTCCCGAGTTTATGAA	
		RPCR2	ATCTTTTTTTGCTTCTATAC	
leuS	CT209	FleuS	TCCCTTGGTCGATCTCCTCAC	519
		RleuS	GGGCATCGCAAAAACGTAAATAGT	
		Alternate:		
		PCRFa	ACAAGACCGGACACTTTGAT	
		PCRRa	AGAACATGCTGTACTGCACT	

Technical Appendix Table 2. Sequence types, allelic profiles, and clinical characteristics of reference and clinical strains of *Chlamydia trachomatis**

		Allele assignment for each locus								
Strain ID†	ST	*glyA*	*mdhC*	*pdhA*	*yhbG*	*pykF*	*lysS*	*leuS*	Region of isolation	Diagnosis/site
L_1/440	1	01	01	03	08	01	04	11	California	LGV
L_1/115	1	01	01	03	08	01	04	11	South Africa	LGV
L_1/1322/p2	1	01	01	03	08	01	04	11	South Africa	
L_1/224	1	01	01	03	08	01	04	11	South Africa	LGV
L_2/54s	1	01	01	03	08	01	04	11	San Francisco	Proctitis
L_2/434	1	01	01	03	08	01	04	11	California	LGV
L_2/25667R	1	01	01	03	08	01	04	11	USA	Proctitis
L_2a/UW396	1	01	01	03	08	01	04	11	Seattle	LGV

		Allele assignment for each locus								
Strain ID†	ST	*glyA*	*mdhC*	*pdhA*	*yhbG*	*pykF*	*lysS*	*leuS*	Region of isolation	Diagnosis/site
L_2b/Ams1	1	01	01	03	08	01	04	11	Netherlands	Proctitis
L_2b/Ams2	1	01	01	03	08	01	04	11	Netherlands	Proctitis
L_2b/Ams3	1	01	01	03	08	01	04	11	Netherlands	Proctitis
L_2b/Ams4	1	01	01	03	08	01	04	11	Netherlands	Proctitis
L_2b/Ams5	1	01	01	03	08	01	04	11	Netherlands	Proctitis
L_2b/Canada1	1	01	01	03	08	01	04	11	Canada	Proctitis
L_2b/Canada2	1	01	01	03	08	01	04	11	Canada	Proctitis
L_2b/CV204	1	01	01	03	08	01	04	11	France	Proctitis
L_2b/LST	1	01	01	03	08	01	04	11	France	Proctitis
L_2b/UCH1	1	01	01	03	08	01	04	11	United Kingdom	Proctitis
L_2b/UCH2	1	01	01	03	08	01	04	11	United Kingdom	Proctitis
L_2b/795	1	01	01	03	08	01	04	11	France	Proctitis
L_2b/8200/07	1	01	01	03	08	01	04	11	Sweden	Proctitis
L_2b/86nl	1	01	01	03	08	01	04	11	Amsterdam	Proctitis
L_2c	1	01	01	03	08	01	04	11	USA	Proctitis
L3/404	1	01	01	03	08	01	04	11	California	LGV
D/84s	2	02	03	03	06	05	04	03	San Francisco	Cervicitis
H/UW4/Cx	3	03	01	03	06	06	04	03	Washington	Cervicitis
A/51t	4	03	03	01	06	03	07	09	Tanzania	Trachoma
H/46nl	5	03	03	02	06	06	08	03	Amsterdam	Cervicitis and vaginal discharge
B/TW5/OT	6	03	03	03	04	03	05	10	Taiwan	Conjunctivitis
I/UW12/Ur	7	03	03	03	06	01	04	03	Washington	Urethritis
K/UW36/Cx	8	03	03	03	06	02	04	03	Washington	Cervicitis
J/UW36/Cx	9	03	03	03	06	02	08	03	Washington	Cervicitis
Ja/UW92	9	03	03	03	06	02	08	03	Washington	Cervicitis
B/53t	10	03	03	03	06	03	04	09	Tanzania	Trachoma
C/TW3/OT	11	03	03	03	06	03	05	07	Taiwan	Conjunctivitis
A/2497	12	03	03	03	06	03	05	09	Tanzania	Trachoma
A/363	12	03	03	03	06	03	05	09	Tanzania	Trachoma
A/48t	12	03	03	03	06	03	05	09	Tanzania	Trachoma
A/5291	12	03	03	03	06	03	05	09	Tanzania	Trachoma
A/59t	12	03	03	03	06	03	05	09	Tanzania	Trachoma
A/7249	12	03	03	03	06	03	05	09	Tanzania	Trachoma
B/TZ1A828/OT	12	03	03	03	06	03	05	09	Tanzania	Trachoma
B/50t	12	03	03	03	06	03	05	09	Tanzania	Trachoma
B/60t	12	03	03	03	06	03	05	09	Tanzania	Trachoma
B/61t	12	03	03	03	06	03	05	09	Tanzania	Trachoma
B/62t	12	03	03	03	06	03	05	09	Tanzania	Trachoma
Ba/52t	12	03	03	03	06	03	05	09	Tanzania	Trachoma
C/32n	13	03	03	03	06	03	06	07	Nepal	Trachoma, TS
C/33n	13	03	03	03	06	03	06	07	Nepal	Trachoma, TS
G/15s	14	03	03	03	06	04	04	08	San Francisco	Proctitis
K/42nl	15	03	03	03	06	06	01	06	Amsterdam	Cervicitis w/ vaginal discharge
K/49nl	15	03	03	03	06	06	01	06	Amsterdam	Cervicitis w/ vaginal discharge
**J/112i**	**15**	**03**	**03**	**03**	**06**	**06**	**01**	**06**	**Indianapolis**	**Urethra**
**J/113i**	**15**	**03**	**03**	**03**	**06**	**06**	**01**	**06**	**Indianapolis**	**Cervix**
K/186i	15	03	03	03	06	06	01	06	Indianapolis	Urethra
K/187i	15	03	03	03	06	06	01	06	Indianapolis	Cervix
J/27s	16	03	03	03	06	06	01	08	San Francisco	Cervicitis/urethritis
E/87e	17	03	03	03	06	06	02	03	Ecuador	Cervicitis
Ba/Apache2	18	03	03	03	06	06	03	09	Arizona	Conjunctivitis
G/SotonG1	19	03	03	03	06	06	04	03	United Kindom	Cervicitis
D/SotonD5	19	03	03	03	06	06	04	03	United Kingdom	Cervicitis
D/SotonD6	19	03	03	03	06	06	04	03	United Kingdom	Cervicitis
D/83s	19	03	03	03	06	06	04	03	San Francisco	Cervicitis
H/18s	19	03	03	03	06	06	04	03	San Francisco	Cervicitis/urethritis
H/40nl	19	03	03	03	06	06	04	03	Amsterdam	Cervicitis
**H/114i**	**19**	**03**	**03**	**03**	**06**	**06**	**04**	**03**	**Indianapolis**	**Urethra**
**H/115i**	**19**	**03**	**03**	**03**	**06**	**06**	**04**	**03**	**Indianapolis**	**Cervix**
I/22p	19	03	03	03	06	06	04	03	Lisbon	Cervicitis/urethritis
J/44nl	19	03	03	03	06	06	04	03	Amsterdam	Cervicitis
D/43nl	20	03	03	03	06	06	04	06	Amsterdam	Cervicitis and vaginal discharge
G/13s	21	03	03	03	06	06	04	08	San Francisco	Proctitis
G/14s	21	03	03	03	06	06	04	08	San Francisco	Proctitis

Strain ID†	ST	Allele assignment for each locus							Region of isolation	Diagnosis/site
		glyA	*mdhC*	*pdhA*	*yhbG*	*pykF*	*lysS*	*leuS*		
A/Sa1	22	03	03	03	06	06	05	02	Saudi Arabia	Trachoma
Ia/UW202	23	03	03	03	06	06	08	03	United Kingdom	Cervicitis
Ia/SotonIa1	23	03	03	03	06	06	08	03	United Kingdom	Cervicitis
Ia/SotonIa3	23	03	03	03	06	06	08	03	United Kingdom	Cervicitis
Ia/57e	23	03	03	03	06	06	08	03	Ecuador	Cervicitis
Ia/94i	23	03	03	03	06	06	08	03	Indianapolis	Urethra
Ia/95i	23	03	03	03	06	06	08	03	Indianapolis	Cervix
Ia/118i	23	03	03	03	06	06	08	03	Indianapolis	Cervix
Ia/119i	23	03	03	03	06	06	08	03	Indianapolis	Urethra
Ia4/177i	23	03	03	03	06	06	08	03	Indianapolis	Urethra
Ia4/180i	23	03	03	03	06	06	08	03	Indianapolis	Cervix
Ia/178i	23	03	03	03	06	06	08	03	Indianapolis	Cervix
Ia/179i	23	03	03	03	06	06	08	03	Indianapolis	Urethra
Ia/183i	23	03	03	03	06	06	08	03	Indianapolis	Urethra
Ia/184i	23	03	03	03	06	06	08	03	Indianapolis	Cervix
D/2s	23	03	03	03	06	06	08	03	San Francisco	Cervicitis/urethritis
Ia/24s	24	03	03	03	06	06	08	08	San Francisco	Cervicitis/urethritis
Ia/25s	24	03	03	03	06	06	08	08	San Francisco	Cervicitis/urethritis
D/UW3/Cx	25	03	03	03	06	07	04	01	Washington	Cervicitis
G/16p	26	03	03	03	07	06	08	05	Lisbon	Cervicitis/urethritis
G/17p	27	03	03	04	06	06	04	03	Lisbon	Cervicitis/urethritis
Ia/23p	28	03	03	04	06	06	04	04	Lisbon	Cervicitis/urethritis
H/21p	29	03	03	04	06	06	08	03	Lisbon	Cervicitis
G/UW57/Cx	30	03	03	05	06	06	04	03	Washington	Cervicitis
H/20p	31	03	03	07	06	06	08	03	Lisbon	Cervicitis/urethritis
F/38nl	32	04	04	03	02	07	04	03	Amsterdam	Cervicitis and vaginal discharge
L_2b/48nl	33	05	02	03	08	01	04	11	Amsterdam	Proctitis
L_2b/85nl	33	05	02	03	08	01	04	11	Amsterdam	Proctitis
F/ICCal3	34	06	03	03	02	07	04	03	United Kingdom	Cervicitis
F/SotonF3	34	06	03	03	02	07	04	03	United Kingdom	Cervicitis
F/SW4	34	06	03	03	02	07	04	03	Sweden	Cervicitis
F/SW5	34	06	03	03	02	07	04	03	Sweden	Cervicitis
F/8p	34	06	03	03	02	07	04	03	Lisbon	Cervicitis/urethritis
F/9p	34	06	03	03	02	07	04	03	Lisbon	Cervicitis/urethritis
F/98i	34	06	03	03	02	07	04	03	Indianapolis	Urethra
F/99i	34	06	03	03	02	07	04	03	Indianapolis	Cervix
F/181i	34	06	03	03	02	07	04	03	Indianapolis	Urethra
F/182i	34	06	03	03	02	07	04	03	Indianapolis	Cervix
F/191i	34	06	03	03	02	07	04	03	Indianapolis	Urethra
F/192i	34	06	03	03	02	07	04	03	Indianapolis	Cervix
D/SotonD1	34	06	03	03	02	07	04	03	California	Cervicitis
**D2/96i**	**34**	**06**	**03**	**03**	**02**	**07**	**04**	**03**	**Indianapolis**	**Urethra**
**D2/97i**	**34**	**06**	**03**	**03**	**02**	**07**	**04**	**03**	**Indianapolis**	**Cervix**
**D2/189i**	**34**	**06**	**03**	**03**	**02**	**07**	**04**	**03**	**Indianapolis**	**Cervix**
**D2/190i**	**34**	**06**	**03**	**03**	**02**	**07**	**04**	**03**	**Indianapolis**	**Urethra**
E/5s	34	06	03	03	02	07	04	03	San Francisco	Cervicitis/urethritis
E/19e	34	06	03	03	02	07	04	03	Ecuador	Cervicitis
Ja/41nl	34	06	03	03	02	07	04	03	Amsterdam	Cervicitis and vaginal discharge
Ja/47nl	34	06	03	03	02	07	04	03	Amsterdam	Cervicitis and vaginal discharge
F/10s	35	06	03	03	02	07	04	08	San Francisco	PID
F/11s	35	06	03	03	02	07	04	08	San Francisco	PID
F/12s	35	06	03	03	02	07	04	08	San Francisco	PID
E/39nl	36	06	03	03	03	07	04	03	Amsterdam	Cervicitis
Da/TW448	37	06	03	03	05	07	04	02	Taiwan	Trachoma
D/3s	38	06	03	06	02	07	04	03	San Francisco	Cervicitis/urethritis
E/Bour	39	06	04	03	02	07	04	03	California	Cervicitis
E/SotonE4	39	06	04	03	02	07	04	03	United Kingdom	Cervicitis
E/SotonE8	39	06	04	03	02	07	04	03	United Kingdom	Cervicitis
E/SW2	39	06	04	03	02	07	04	03	Sweden	Urethritis
E/SW3	39	06	04	03	02	07	04	03	Sweden	Cervicitis
E/6p	39	06	04	03	02	07	04	03	Lisbon	Cervicitis
E/7p	39	06	04	03	02	07	04	03	Lisbon	Cervicitis
E/28e	39	06	04	03	02	07	04	03	Ecuador	Cervicitis
E/45nl	39	06	04	03	02	07	04	03	Amsterdam	Cervicitis

		Allele assignment for each locus								
Strain ID†	ST	*glyA*	*mdhC*	*pdhA*	*yhbG*	*pykF*	*lysS*	*leuS*	Region of isolation	Diagnosis/site
E/55e	39	06	04	03	02	07	04	03	Ecuador	Cervicitis
E/56e	39	06	04	03	02	07	04	03	Ecuador	Cervicitis
E/58t	39	06	04	03	02	07	04	03	Tanzania	Conjunctivitis
E/88i	39	06	04	03	02	07	04	03	Indianapolis	Cervix
E/89i	39	06	04	03	02	07	04	03	Indianapolis	Urethra
E/102i	39	06	04	03	02	07	04	03	Indianapolis	Urethra
E/103i	39	06	04	03	02	07	04	03	Indianapolis	Cervix
E/106i	39	06	04	03	02	07	04	03	Indianapolis	Urethra
E/107i	39	06	04	03	02	07	04	03	Indianapolis	Cervix
E/108i	39	06	04	03	02	07	04	03	Indianapolis	Cervix
E/109i	39	06	04	03	02	07	04	03	Indianapolis	Urethra
E/110i	39	06	04	03	02	07	04	03	Indianapolis	Cervix
E/111i	39	06	04	03	02	07	04	03	Indianapolis	Urethra
E/116i	39	06	04	03	02	07	04	03	Indianapolis	Urethra
E/117i	39	06	04	03	02	07	04	03	Indianapolis	Urethra (female)
E6/120i	39	06	04	03	02	07	04	03	Indianapolis	Cervix
E6/121i	39	06	04	03	02	07	04	03	Indianapolis	Urethra
E/171i	39	06	04	03	02	07	04	03	Indianapolis	Urethra
E/172i	39	06	04	03	02	07	04	03	Indianapolis	Cervix
E/150	39	06	04	03	02	07	04	03	Seattle	Proctitis
E/11023	39	06	04	03	02	07	04	03	Seattle	Cervicitis
E/4s	40	06	04	03	03	07	04	03	San Francisco	Cervicitis/urethritis
Ja/26s	41	06	04	03	05	06	04	08	San Francisco	Cervicitis/urethritis
C/31n	42	07	03	03	01	03	05	07	Nepal	Trachoma, TI
C/35n	43	07	03	03	06	03	05	07	Nepal	Trachoma, TI
C/1n	44	07	03	03	06	03	06	07	Nepal	Trachoma, TI
C/29n	44	07	03	03	06	03	06	07	Nepal	Trachoma, TI
C/30n	44	07	03	03	06	03	06	07	Nepal	Trachoma, TI
C/34n	44	07	03	03	06	03	06	07	Nepal	Trachoma, TI
C/36n	44	07	03	03	06	03	06	07	Nepal	Trachoma, TI
C/37n	44	07	03	03	06	03	06	07	Nepal	Trachoma, TI
**D1/90i**	**45**	**03**	**03**	**03**	**02**	**07**	**04**	**03**	**Indianapolis**	**Urethra**
**D1/91i**	**45**	**03**	**03**	**03**	**02**	**07**	**04**	**03**	**Indianapolis**	**Cervix**
**E/92i**	**46**	**06**	**03**	**03**	**05**	**07**	**04**	**08**	**Indianapolis**	**Urethra**
**E/93i**	**46**	**06**	**03**	**03**	**05**	**07**	**04**	**08**	**Indianapolis**	**Cervix**
**E/104i**	**46**	**06**	**03**	**03**	**05**	**07**	**04**	**08**	**Indianapolis**	**Urethra**
**E/105i**	**46**	**06**	**03**	**03**	**05**	**07**	**04**	**08**	**Indianapolis**	**Cervix**
**E/173i**	**46**	**06**	**03**	**03**	**05**	**07**	**04**	**08**	**Indianapolis**	**Cervix**
**E/174i**	**46**	**06**	**03**	**03**	**05**	**07**	**04**	**08**	**Indianapolis**	**Urethra**
**E/185i**	**46**	**06**	**03**	**03**	**05**	**07**	**04**	**08**	**Indianapolis**	**Urethra**
**E/188i**	**46**	**06**	**03**	**03**	**05**	**07**	**04**	**08**	**Indianapolis**	**Cervix**
E/100i	47	06	04	03	05	06	04	03	Indianapolis	Cervix
E/101i	47	06	04	03	05	06	04	03	Indianapolis	Urethra
D/EC	48	03	03	03	06	06	04	01	Montana	
D/LC	48	03	03	03	06	06	04	01	Montana	
K/SotonK1	49	03	03	03	06	06	04	13	United Kingdom	Cervicitis
B/Jali20	50	03	03	03	06	06	05	07	Gambia	Trachoma
G/11074	51	03	03	03	06	06	09	03	Seattle	Proctitis
G/9301	51	03	03	03	06	06	09	03	Seattle	Urethritis
G/9768	51	03	03	03	06	06	09	03	Seattle	Proctitis
G/11222	52	03	03	05	06	06	01	03	Seattle	Cervicitis
A/HAR13	53	03	05	03	06	03	05	07	Tunisia	Trachoma
F/1	54	06	03	03	02	06	04	03	USA	Cervicitis
F4/175i	55	06	03	03	02	07	04	12	Indianapolis	Cervix
F4/176i	55	06	03	03	02	07	04	12	Indianapolis	Urethra
J/151s	56	06	03	03	05	07	04	03	USA	Cervicitis

*Gray shading indicates reference strains. Boldface indicates putative recombinant strains. Paired strains in boxes are from heterosexual dyads; urethral strains are from male patients, except as noted. GenBank accession nos. for *ompA* variants: D1 FJ261929; D2 FJ261926; Ia4 FJ261941.1; E6 FJ261948.1; F4 FJ261936. LGV, lymphogranuloma venereum; TS trachomatous scarring; PID, pelvic inflammatory disease; TI, trachomatous inflammation severe; ST, sequence type.

†Strain ID, first letter refers to the *ompA* genotype; the number after the dash represents the ID# of the clinical strain; the small case letter after the number denotes the geographic region from which the sample was obtained: e, Ecuador; i, Indianapolis, Indiana; nl, the Netherlands; n, Nepal; p, Portugal; s, San Francisco**.**

Technical Appendix Table 3. Characteristics of alleles for each locus for reference and clinical strains of *Chlamydia trachomatis*

Gene locus	No. alleles	Length, bp	No. polymorphic sites	Average pairwise distance	Average dS	Average dN
glyA	7	522	5	0.0033658	0.0018735	0.0014918
mdhC	5	519	4	0.0015044	0.0010161	0.0004882
pdhA	7	549	6	0.0001887	0.0000567	0.0001320
yhbG	8	504	21	0.0125564	0.0116917	0.0008647
pykF	7	525	7	0.0032766	0.0013897	0.0018869
lysS	9	576	10	0.0015043	0.0010815	0.0004228
leuS	13	519	12	0.0031572	0.0008716	0.0022855
Total	56	3,714	65	0.0036505	0.0025687	0.0010817
